# Quantifying beryllium concentrations in plant shoots from forest ecosystems using cation‐exchange chromatography and quadrupole ICP‐MS

**DOI:** 10.1002/ansa.202000036

**Published:** 2020-06-08

**Authors:** David Uhlig, Tatiana Goldberg, Daniel A. Frick, Friedhelm von Blanckenburg

**Affiliations:** ^1^ Earth Surface Geochemistry GFZ German Research Centre for Geosciences Telegrafenberg Potsdam 14473 Germany; ^2^ Institute of Geological Sciences Freie Universität Berlin Berlin Germany

**Keywords:** bioindicator, discrimination, matrix effects, non‐polluted ecosystems, toxicity

## Abstract

Beryllium (Be) is known to be one of the most toxic elements but at the same time exerts a stimulating effect on plant growth. Despite this contradiction, little is known about the Be metabolism in living organisms, partially because of the low amounts present and because the analysis of Be in plants by ICP‐MS remains challenging. The challenges arise from the complex organic matrix, the low abundance of Be relative to the other plant essential elements, and the matrix effects resulting thereof in the plasma. To address these challenges, we developed and evaluated a new method for Be concentration analysis in plant material. Key is the quantitative separation of Be from the other matrix elements by cation‐exchange chromatography. The new method was verified by processing seven reference materials representing different plant matrices yielding a long‐term reproducibility of 16% (RSD). Applying the method, Be concentrations in tree, shrub, bush, and grass samples grown in non‐polluted ecosystems from four temperate forests and a tropical rainforest were measured. The Be concentrations in different plant organs range from 0.01 to 63 ng/g that suggest a natural baseline for Be concentrations of 52 ng/g (95 percentile of non‐woody tissue) that may serve as bioindicator for Be pollution in the environment. Comparison of Be concentrations in plants with the soil's biologically available fraction revealed that Be is discriminated from uptake into shoots and thus can be considered as non‐essential.

## 1 INTRODUCTION

The alkaline earth metal element beryllium (Be) occurs in trace amounts in environmental material.^1^ Whereas the Be concentration of the upper continental crust is about 3 μg/g (ref. ^2^) the Be concentration of river water is in the low ng/L range (eg, see refs. ^1^, ^3^, ^4^, ^5^). Beryllium is known to be one of the most toxic elements, and beryllium concentrations reported in non‐crop plants range from as low as 0.0013 μg/g up to 2.0 μg/g.^1^, ^3^, ^6^, ^7^, ^8^, ^9^, ^10^, ^11^, ^12^, ^13^, ^14^, ^15^ Because Be also occurs in coal at the lower μg/g level, Be contributes to air pollution through the combustion of fossil fuel and appears at elevated levels in polluted environments. In polluted environments, Be accumulating on macrohydrophytes reaches concentrations of up to 136.6 μg/g.^16^ However, in polluted environments bulk feeds and cereal grains are only slightly enriched in Be concentrations.^3^ To humans, Be is highly carcinogenic,^17^, ^18^ can trigger a debilitating and potential fatal lung disease known as berylliosis or chronic beryllium disease,^19^, ^20^, ^21^ and can cause inflammation and sensitization reactions when exposed dermal or pulmonal.^22^ Beryllium can also be toxic to plants (eg, see refs. ^23^, ^24^, ^25^) because Be reduces plant growth by reducing seed germination^24^ of, for example, oat and potato tubers,^26^ and by reducing the uptake of plant essential micronutrients.^27^ Given that Be replaces magnesium (Mg) in plants, Be can also cause symptoms of magnesium deficiency.^23^, ^28^, ^29^ Moreover, Be reduces root length in squash by up to 46%.^24^ Finally, Be inhibits the activity of phosphoglucomutase and limits the conversion of glucose‐1‐phosphate to glucose‐6‐phosphate.^30^

The question is whether Be also offers beneficial effects to plants, or whether plants avoid Be uptake to prevent toxicity. Early studies showed that Be can stimulate plant growth when substituting for Mg.^28^ Although Mg is a central atom in the porphyrin ring of the chlorophyll molecule,^31^, ^32^ Be is not involved in chlorophyll production.^28^ Also, Be fertilization experiments on food crops showed an increase in phosphorus (P) uptake and a simultaneous decrease in Ca and Mg uptake.^33^ This lack of consensus demonstrates that the uptake processes of Be by plants remain poorly understood. Beryllium may be transported from the root hair into the xylem by both the apoplast and the symplast pathway.^27^ In either case, Be is assumed to be transported through the plasma membrane by proton pumps, co‐transporter and anti‐transporter, and ionic channels.^30^ To date, it is assumed that within the plant Be is not readily translocated from roots to the aboveground plant tissue. Culture experiments by Romney and Childress^33^ showed that Be concentrations in plant organs are highest in roots. Kaplan et al^24^ showed that 97% of Be remained in roots meaning that only 3% of utilized Be are translocated to aboveground plant tissue. However, in light of today's knowledge, these results are highly questionable. In these experiments, to enable analysis, Be was highly enriched in the nutrient solution, up to a level of about 1‐10 μg/g. Such high Be concentrations exceed the theoretical solubility limit of Be(OH)_2_ in pH neutral aqueous solution of about 1.4 ng/g by 3 to 4 orders of magnitude.^34^, ^35^ Hence, precipitation of Be likely occurred entailing its adsorption onto the root's surfaces.

Given how little is known about Be cycling in plants, the use of Be concentrations in plants as a bioindicator of Be pollution to the environment^36^ is still not possible. In this contribution, we explore the natural cycling of Be in forest ecosystems by determining Be concentrations in aboveground woody and non‐woody plant tissue and in the biologically available pool in the soil. To this end, a prerequisite is the ability to precisely determine very low concentrations of Be in plant material. This determination is challenging, given that the concentrations of the plant´s macronutrients (K, Ca, Mg, and P) exceed those of Be by up to six orders of magnitude. Thus, ICP‐MS measurements will most likely be affected by matrix effects occurring during plasma ionization and ion extraction.

Strategies to circumvent these matrix effects are well‐known, for example, by dilution, standard addition or matrix matching. However, all of these methods have disadvantages: dilution decreases the signal intensity of the trace element of interest, standard addition requires a large sample amount, and matrix matching is complicated given that the plant's chemical composition depends on plant type. Early methods employed (a) the complexation of Be with colorimetric and fluorometric reagents and (b) the separation of Be from matrix elements. (c) More recent studies made advantage of modern ICP‐based techniques. Regarding (a), the most sensitive complexing reagent for Be is morin (2′,4′,3,5,7‐pentahydroxyflavone). This method reaches a limit of detection of 0.02 ng/g.^1^ The disadvantage of such colorimetric and fluorometric methods is the impurity of the complexing reagent. Regarding (b), quantitative precipitation of Be occurs at pH 8.5 in ammonia media only if fluoride, silica, phosphate, and organic matter are absent (eg, see ref. ^37^). As this condition is not met for digested plant material, another extraction method was suggested using acetylacetone/MIBK (methylisobutylketone) in presence of EDTA followed by a re‐extraction with nitric acid and analysis by flameless atomic absorption. This method achieved a limit of detection of 1.5 ng/g.^9^ Finally, a two‐step ion chromatography method was developed by Hayashibe et al^38^ to eliminate spectral interferences during analysis by AAS. In this method, Be is purified in biological material from matrix elements by using cation‐exchange resin (BioRad™ AG® 50W‐X8, 100‐200 mesh) in hydrogen form and anion‐exchange resin (BioRad™ AG® 1‐X8, 100‐200 mesh) in chloride form. Yet, this method consumes a high volume of acid making it prone to substantial blank contribution. Regarding (c), initial US Environmental Protection Agency (EPA) standard methods relied on ICP‐AES, AAS, and ICP‐OES for Be analyses with limits of detection from 0.2 to 5 ng/g for various sample matrices excluding vegetation.^39^, ^40^ With the advancement of analytical instruments Be is nowadays measured by quadrupole inductively coupled plasma mass spectrometry (Q‐ICP‐MS) with a limit of detection in the order of 1 pg/g (eg, see ref. ^12^). However, to date it has not been systematically explored whether Be is best purified from matrix elements prior to Q‐ICP‐MS analyses or can be analyzed directly in the digest.

Here, we developed a new single‐step cation‐exchange chromatography method to separate Be from matrix elements which allows us to test whether a purification of Be from matrix elements prior to Q‐ICP‐MS analysis is required. This method was applied to seven reference materials representing different plant matrices and to grass as well as woody and non‐woody tissue of trees, shrubs and bushes from four temperate forest ecosystems and a tropical rainforest.

## 2 STUDY SITES

Our five well‐studied field sites are located in the Southern Sierra Critical Zone Observatory (SSCZO, USA, here termed SN, eg, see ref. ^41^), the Conventwald (Black Forest, Germany, here termed CON, eg, see ref. ^42^), Mitterfels (Bavarian Forest, Germany, here termed MIT, eg, see ref. ^42^), in the Hakgala Mountain (central Highlands of Sri Lanka, here termed SL, eg, see ref. ^43^), the French Jura Mountains (here termed JU, eg, see ref. ^44^). The study site´s main characteristics are summarized in Table 1. In principle, the study sites differ in climate and thus flora, lithology, and soil type. Whereas temperate forest ecosystems occur in SN, CON, MIT, JU, a tropical rainforest is situated in SL. The vegetation cover differs substantially among study sites. SN is characterized by a mixed conifer forest comprising the main tree species *Pinus ponderosa* (Ponderosa Pine), *Pinus jeffreyi* (Jeffrey Pine), *Pinus lambertiana* (Sugar Pine), *Calocedrus decurrens* (Incense‐Cedar), *Abies concolor* (White Fir), and shrubs consisting of *Chamaebatia foliolosa* (Mountain Misery), *Ceanothus cordulatus* (Mountain Whitethorn), and *Arctostaphylos manzanita* (Manzanita). CON and MIT are covered by a mixed deciduous forest of the main tree species *Fagus sylvatica* (European beech) and *Picea abies* (Norway spruce). SL hosts 97 tree species of which 62 are endemic. The most common tree species are: *Allophylus varians*, *Cinnamonum ovalifolium*, *Eugenia mabaeoides*, *Memecylon parvifolium*, *Michelia nilagirica*, *Neolitsea fuscata*, *Psychotria bisulcata*, *Semecarpus coracea*, *Symplocos loha*, *Syzygium revolutum*, and *Syzygium rotundifolium*.^45^ At JU, a deciduous forest dominates under 800 m.a.s.l. while an evergreen coniferous forest prevails above 800 m.a.s.l.. Tree species gradually shift from oak (*Quercus robur, Quercus pedonculata*) in the lowlands to conifers (*Picea abies, Abies alba*) at higher altitude.

**TABLE 1:** Main characteristics of study sites

Study site	Longitude^a^	Latitude^a^	Altitude (m.a.s.l.)	MAT (°C)	MAP (mm)	Soil type	Lithology
Conventwald (CON)	48°1.20222′N	7°57.93996′E	733 – 863	6.8 ^b^	1749 ^b^	Dystric Cambisol ^d^	Paragneiss
Mitterfels (MIT)	48°58.54860′N	12°52.49388′E	985 – 1037	5.5 ^b^	1580 ^b^	Dystric Cambisol ^d^	Paragneiss
Sierra Nevada (SN)	37°3.06330′N	‐119°12.32946′E	1479 – 2113	7.8 ^b^	750‐2000 ^c^	Entisol, Inceptisol	Granodiorite
Sri Lanka (SL)	6°55.75380′N	80°49.10040′E	1753	16 ^b^	2013	Ultisol	Charnockite
Jura (JU)							
Dard	46°41.592′N	5°38.958′E	512	10.2 ^b^	1090 ^b^	Brown Luvisol ^b^	Limestone
Dahon	47° 4.914′N	6° 14.382′E	628	10.2 ^b^	1090 ^b^	Brown Luvisol ^b^	Limestone
Dessoubre	47° 9.192′N	6° 36.060′E	640	10.2 ^b^	1090 ^b^	Brown Luvisol ^b^	Limestone
Lison	46° 57.846′N	6° 1.362′E	662	10.2 ^b^	1090 ^b^	Brown Luvisol ^b^	Limestone
Saine	46° 40.320′N	6° 4.566′E	1004	7.5 ^b^	1500 ^b^	Humocalcic ^b^	Limestone
Doubs	46° 41.178′N	6° 12.390′E	1182	7.5 ^b^	1500 ^b^	Humocalcic ^b^	Limestone

a Catchment coordinates according to WGS84.

b Data origin: CON: Forstliche Versuchsanstalt Baden‐Wuerttemberg (FVA), MIT: Bayerische Landesanstalt für Wald und Forstwirtschaft (LWF), SN^69^, SL^43^, JU.^44^

c Data from ref. ^70^.

d Soil type according to: CON and MIT: World Reference Base for Soil Resources, SN and SL: USDA Soil Taxonomy.

## 3 MATERIALS AND METHODS

### 3.1 Sampling

#### 3.1.1 Vegetation

At SN, freshly fallen plant tissue was sampled from the forest floor. Leaves were sampled from the shrub species *Ceanothus cordulatus* and *Arctostaphylos manzanita*, needles and twigs were sampled from the tree species *Pinus jeffreyi*, and *Abies concolor*, and bark was sampled from *Abies concolor*. At CON and MIT living wood, leaves and needles were sampled from the tree species *Fagus sylvatica* and *Picea abies*. Living wood was sampled using an increment borer (diameter of 4.3 mm). Leaves and needles were sampled from young branches closest to the forest floor. At SL, leaves were sampled from the shrub species *Maesa indica, Cestrum aurantiacum*, and *Eurya japonica*, from the grass species *Arundinaria debilis*, and from the tree species *Neolitsea fuscata*. Leaves and twigs were sampled from the tree species *Ixora calycina, Ilex walkeri*, and *Ixora coccinea*. At JU, living wood was sampled from *Picea, Fagus, Quercus*, and *Abies alba* by using an increment borer, bark from *Abies alba* by peeling off, and grass from *Poaceae* by cutting the aboveground part.

#### 3.1.2 Soil and saprolite

At SN, bulk soil and saprolite were sampled from the soil‐saprolite roadcut profile called the “Balsam Profile”, which covers deep saprolite up to 600 cm depth. At CON and MIT, regolith – defined to comprise mobile soil and immobile saprolite – was sampled at depth increments of 0.2 m from a 3 m deep trench. Regolith beyond 3 m depth was sampled from 20 m (CON) and 30 m (MIT) deep drill cores (see ref.^42^ for details) as composite samples collected from about 0.5 m to about 1 m core sections.

### 3.2 Laboratory, instrumentation, and reagents

Analytical procedures were carried out at the Helmholtz Laboratory for the Geochemistry of the Earth Surface (HELGES^46^). Microwave assisted sample digestion was performed in an ISO 6 class metal‐free clean laboratory using a Milestone microwave digestion system (MLS Start, Germany) equipped with a temperature and pressure sensor and a sample carousel holding 10 PTFE vessels with a capacity of 100 mL. Cation‐exchange chromatography and sample evaporation were carried out in ISO 4 laminar flow wet benches (Arias, Germany). Beryllium concentration measurements with and without purification from matrix elements were done on a quadrupole inductively coupled plasma mass spectrometer (iCAP‐Q, Thermo Fischer Scientific, Germany) equipped with a ESI quartz glass spray cyclonic chamber, ESI PFA‐MicroFlow nebulizer (100 μL/min), prep FAST system (ESI fast DX, 1.5 mL loop), and skimmer cone with a 2.8 mm high sensitivity insert.

Concentrated HNO_3_ and HCl used in this study were sub‐boiled in quartz glass and diluted to the required molarity using deionized water (mQ‐H_2_O, 18.2 MΩ cm, TOC < 3 ng/g from Merck Millipore, Germany). The 47‐51% HF (Normatom, VWR Chemicals), 30% H_2_O_2_ (Merck Millipore, Germany), and sulfonated polystyrene cation‐exchange resin (AG® 50W‐X12, 200‐400 mesh, 2.1 meq/mL, BioRad™, USA) were of analytical grade. Mono‐elemental solutions of Certipur® grade of the elements Be, Li, Al, Ti with a concentration of 1000 mg/L (Merck Millipore, Germany) were used to prepare standards for external calibration and internal standards and used for doping tests. Labware, such as test tubes and pipette tips, were soaked at room temperature at least overnight each in 1 M HCl, 1 M HNO_3_, and mQ‐H_2_O prior to use. Similarly, PFA vials were soaked at 110°C at least overnight each in 6 M HCl, 7.5 M HNO_3_, and mQ‐H_2_O prior to use.

### 3.3 Decomposition of plant material

Prior to plant digestion PTFE vessels were cleaned with 14 mL of 7.5 M HNO_3_ using the same microwave procedure as for plant digestion. A total of 100‐500 mg (dry mass) of plant material were weighed directly into PTFE vessels. Higher sample amounts lead to the formation of an excessive gas volume. Thus, for a total amount of ∼1000 mg (dry mass), the sample was initially split into two parts and subsequently combined. Based on Sucharová and Suchara,^12^ a mixture containing 5 mL of mQ‐H_2_O, 4 mL of ∼14 M HNO_3_, and 3 mL of 30% H_2_O_2_ was used to digest plant material. This mixture was added to the samples and led at room temperature to react for 30 min. The microwave procedure was then run by gradually heating for 12.5 min to 200°C, holding the temperature for 17.5 min and venting for another 18 min to allow cooling and degassing. Digested plant material was transferred quantitatively into 22 mL PFA vials and evaporated to dryness at sub‐boiling temperature (110°C) on a hotplate coated with Teflon®. To remove biogenic silica, a 3:1 mixture containing up to 3 mL of 47‐51% HF and 1 mL of ∼14 M HNO_3_ was added and heated at 110°C overnight. After evaporation to dryness at 110°C, 6 mL of concentrated *aqua regia* was added, and heated a second time overnight at 170°C. After evaporation again to dryness at 110°C samples were re‐dissolved in 5 mL of 0.3 M HNO_3_. For most reference materials, an aliquot prior to cation‐exchange chromatography was taken and diluted 100 times to measure Be concentration without purification to assess the matrix effects.

### 3.4 Cation‐exchange chromatography

According to a recent review on microwave‐assisted digestion methods with subsequent ICP‐MS analysis,^47^ ICP‐MS measurements require a low amount of total dissolved solids of <0.1% to avoid the deposition of salts on cones of the ICP‐MS interface, and dissolved organic carbon (DOC) should be minimized because ICP‐based techniques present a poor tolerance to dissolved carbon. Easily ionizable elements such as K, Ca, Mg, Na can cause a signal suppression of the analyte of interest having a higher ionization potential (eg, see refs. ^48^, ^49^), and carbon can cause a sensitivity increase on the analyte of interest (see refs.^50^, ^51^, ^52^). The residual carbon content (RCC) – defined as the percentage of dissolved carbon to the samples´ original carbon content (m/m) – after wet digestion ranges typically between 1% and 30%^47^ and depends on the molarity of nitric acid used during microwave digestion. At 3 M HNO_3_, RCC is >48%; using 7 M HNO_3_ RCC is <12%.^53^ RCC also depends on the temperature (typically up to 250°C, see refs.^47^, ^54^). Given the lower temperature (200°C) and the low molarity (3.5 M) of the digestion acid used in this study, we suspect that substantial RCC will be present in our digests.

**FIGURE 1:**
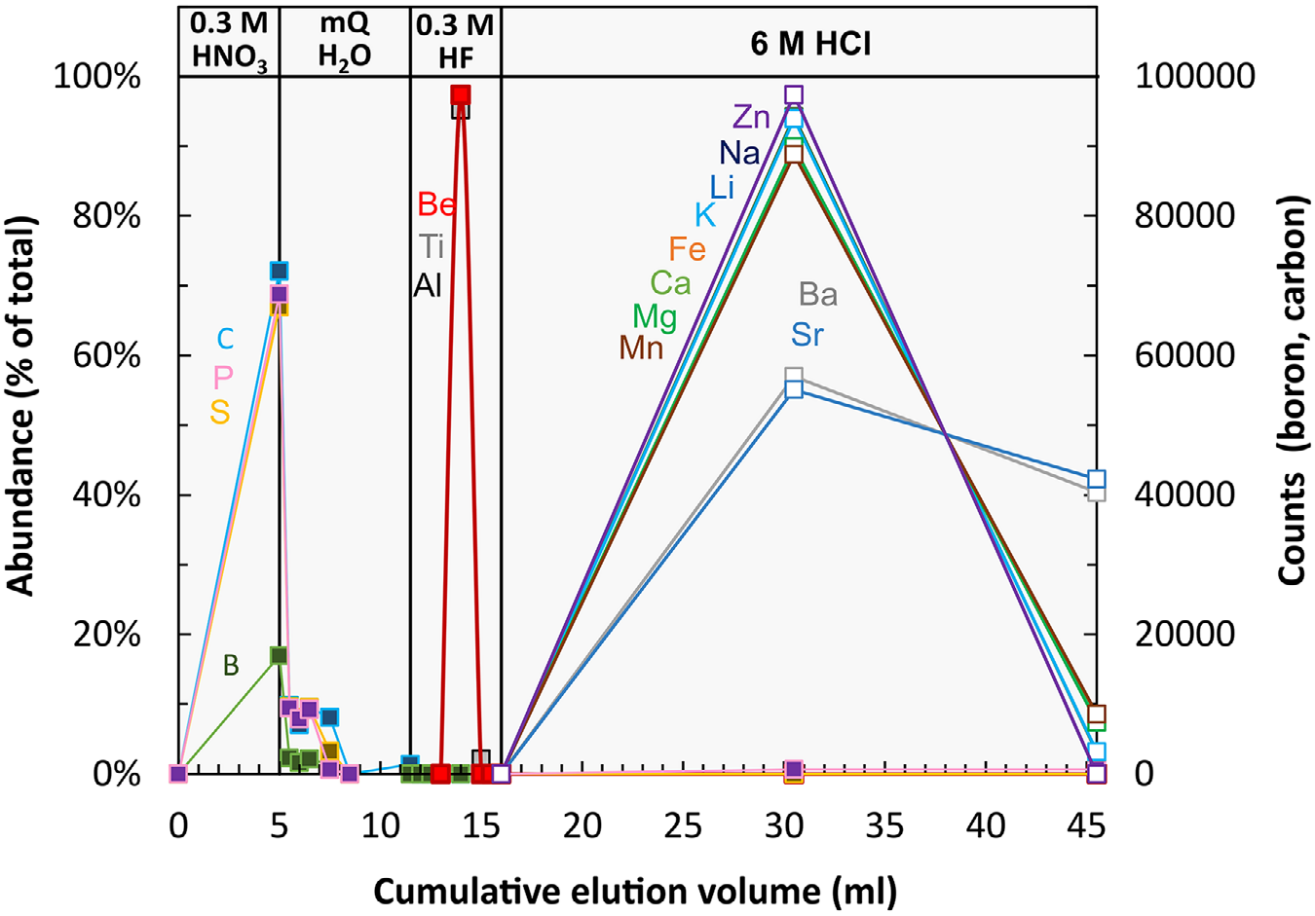
Elution curves of selected elements for NIST SRM 1515 Apple leaves spiked with 5 μg of Be and Li. Note counts for boron and carbon are acid blank‐corrected

To separate matrix elements and RCC from Be in digested plant material, cation‐exchange chromatography was employed. For this purpose, disposable polypropylene 50 mL Pasteur pipettes (BRAND®, Germany) were turned up‐side down, equipped with UHMW‐PE frits of 20 μm pore size, and packed with 3 mL of AG® 50W‐X12 200‐400 mesh resin (BioRad™, USA). In order to achieve a high aspect ratio, the inner diameter of the columns was 6.4 mm and the resin bed was 92 mm in length (in mQ‐H_2_O). Elution curves were obtained using NIST SRM 1515 Apple leaves and element concentrations of eluants were measured by inductively coupled plasma optical emission spectrometry (ICP‐OES, Varian 720ES). Due to the low concentration of Be and Li in NIST SRM 1515 Apple leaves the sample was spiked with Be and Li (each 5 μg) to ensure detection above the background and hence to better assess the recovery of Be and removal of Li on an aliquot representative for unknown samples. Elution curves are shown in Figure 1 and the full elution protocol is provided in Table 2.

**TABLE 2:** Elution protocol for a single‐step separation of Be from matrix elements

Reagent	Volume (mL)	Procedure
0.3 M HNO_3_	6	Precondition
0.3 M HNO_3_	5‐10	Load sample
mQ‐H_2_O	0.5	Elution of P, S, C, B
mQ‐H_2_O	0.5	
mQ‐H_2_O	0.5	
mQ‐H_2_O	1	
mQ‐H_2_O	1	
mQ‐H_2_O	3	
0.3 M HF	0.5	Collection of Be (co‐elution with Al, Ti)
0.3 M HF	0.5	
0.3 M HF	0.5	
0.3 M HF	1	
0.3 M HF	1	
0.3 M HF	1	
6 M HCl	30	Cleaning
6 M HCl	30	
mQ‐H_2_O	30	Remove 6 M HCl

The resin was conditioned with 6 mL of 0.3 M HNO_3_ (two column volumes) followed by loading 5–10 mL of re‐dissolved sample onto the columns. The sample was washed into the column with 6.5 mL of deionized water with the first 1.5 mL incrementally in steps of 0.5 mL. Finally, Be was eluted with 5.5 mL of 0.3 M HF with the first 1.5 mL incrementally in steps of 0.5 mL. Prior to column re‐use, the remaining matrix elements were eluted with 60 mL of 6 M HCl (20 column volumes). Pre‐ and post‐Be cuts were not routinely taken as Be breakthrough was prevented by using <10% of the resin capacity. Also, minor shifts in the elution peak would not be quantifiable in these pre‐ or post‐Be cuts. After cation‐exchange chromatography, the Be fraction was evaporated to dryness at 110°C and re‐dissolved in 4 mL of 0.3 M HNO_3_ for Q‐ICP‐MS analyses.

An alternative Be separation scheme frequently used for separation of very small amounts of cosmogenic ^10^Be uses oxalic acid and 0.5 M HCl (or alternatively 1 M HNO_3_) instead of 0.3 M HNO_3_ and 0.3 M HF to separate Be from matrix elements.^55^ This method was designed for rocks, minerals, and water samples and has the advantage that trivalent cations such as Fe^3+^, Al^3+^, and Ti^3+^ are eluted first as metal oxalates while Be is retained and separated from Na and K with 0.5 M HCl. However, a non‐negligible fraction of the Mg elution peak, and small fractions of the K and Ca peak overlap with the Be peak (later separated by hydroxide precipitation). As these are the elements most abundant in plant materials, we decided not to apply this separation scheme.

### 3.5 Be concentration of plants measured with Q‐ICP‐MS

To monitor and correct for sensitivity fluctuations in ICP‐MS measurements, internal standards of similar mass, chemical, and physical behavior of the analyte of interest should be used. For the analyte Be, the best internal standards are boron (B) and lithium (Li). Even though both elements are quantitatively removed by cation‐exchange chromatography, boron causes memory effects in the introduction system and thus requires long rinse times. To avoid this, Li (5 ng/g) was used as internal standard for the purified samples. For Be measurements on aliquots of digested reference materials without purification rhodium (5 ng/g) was used as internal standard. Beryllium concentration measurements were carried out in the STD mode with an external calibration. The Be concentration of the calibration standards ranged from 0.001 to 10 ng/g. Instrumental operating parameters are listed in Table 3.

**TABLE 3:** Instrumental operating parameters for ^9^Be analyses with Q‐ICP‐MS (iCAP, Thermo Fischer Scientific)

Mass spectrometer settings	
RF power	1550 W
Cool gas flow rate	14 L/min
Auxiliary gas flow rate	0.8 L/min
Nebuliser gas flow rate	1.19 L/min
Nebulizer flow rate	100 μL/min
Peristaltic pump speed	40 rpm
Dwell time	40 ms
Runs/sweeps	3/100
Isotopes	^7^Li, ^9^Be, ^27^Al, ^48^Ti, ^13^C, (^103^Rh)

### 3.6 Element concentration of plants measured with ICP‐OES

The chemical composition of plant samples from SN, CON, and MIT were previously measured and reported by Schuessler et al^56^ and Uhlig et al.^57^ In brief, major and trace element concentrations were analyzed by inductively coupled plasma optical emission spectrometry (ICP‐OES, Varian 720ES) with relative uncertainties of 10% based on full analytical replicates of NIST SRM 1515 Apple leaves.

### 3.7 Analysis of the biologically available fraction

The procedure of a sequential extraction method depends on the element of interest. To extract the biologically available fraction of (a) P, (b) Be, and (c) the elements K, Ca, Mg, Na, Al, Fe, and Mn, three different methods were applied. Whereas the Be extraction method is described in detail in Uhlig and von Blanckenburg ^58^, the methods for P and the other elements are described in Wittmann et al^42^. All methods are briefly summarized here.

The biologically available fraction of P was analyzed at the University of Bonn (INRES) by modifying the Hedley fractionation method from Tiessen and Moir^59^ by extracting P from 0.5 g bulk soil (dried, sieved to <2 mm) with 30 mL of 1 M HCl for 16 h on an overhead shaker, followed by centrifugation and filtration through ashless quantitative paper filters. The concentration of inorganic‐bound P (P_i_) was determined by the molybdenum‐blue method^60^ and total P by ICP‐OES (Ultima 2, HORIBA Jobin Yvon, Longjumeau, France); organic‐bound P (P_o_) was calculated as the difference of total P and P_i_.

The biologically available fraction of Be is represented by amorphous and poorly crystalline oxides extracted from 0.5 g bulk soil (dried, sieved to <63 μm) with 10 mL of 0.5 M HCl by mild shaking for 24 h and centrifuged for 20 min at 4200 rpm. The supernatants were pipetted off, treated with concentrated acid mixtures (HF, HCl, HNO_3_) and re‐dissolved prior to concentration analyses. Beryllium concentrations were measured with ICP‐OES in 0.3 M HNO_3_ and matrix matched calibration standards with a relative uncertainty of 5% based on accuracy of repeat analyses of the international reference materials GA (granite, CNRS) and RGM‐1 (rhyolite, USGS).

The biologically available fraction of the other elements is represented by the easily exchangeable fraction extracted from 0.5 g (SN samples) or 2 g (CON and MIT samples) bulk soil (dried, sieved to <2 mm) with 14 mL of 1 M NH_4_OAc at neutral pH for 2 h by mild shaking at 7 rpm, centrifuged at 4200 rpm for 30 min. The supernatant was pipetted off into a syringe and filtered through a 0.2 μm acetate filter, treated with concentrated acid mixtures (HF, HCl, HNO_3_) and re‐dissolved prior to concentration analyses. Element concentrations were measured with ICP‐OES following the procedure described in Schuessler et al,^61^ with relative uncertainties better than 5% (Al, Ca, Fe, Mn, Na) and 10% (K, Mg) based on repeat analyses of the reference materials SLRS‐5 (river water, NRC CNRC), SRM 1640a (river water, NIST), and M212 (USGS) and synthetic in‐house standards.

## 4 RESULTS

### 4.1 Chromatographic separation of beryllium

The recovery of Be after cation‐exchange chromatography was found to be 100%. Beryllium can be quantitatively separated from the matrix elements phosphorus (P), sulfur (S), carbon (C), and boron (B) because these elements breakthrough the cation‐exchange resin (Figure 1) in anionic or charge‐neutral form. The removal of residual carbon is of particular importance as carbon is known to increase the sensitivity of elements with a lower first ionization potential, for example, Be (9.3 eV), than carbon (11.26 eV) during ICP‐MS analyses (eg, see refs. ^50^, ^62^, ^63^). Beryllium is also quantitatively separated from the macronutrients K, Ca, Mg as well as most micronutrients, plant beneficial elements, and Li, which are eluted with 6 M HCl (Figure 1) from the resin during the cleaning step prior to resin re‐use.

**FIGURE 2:**
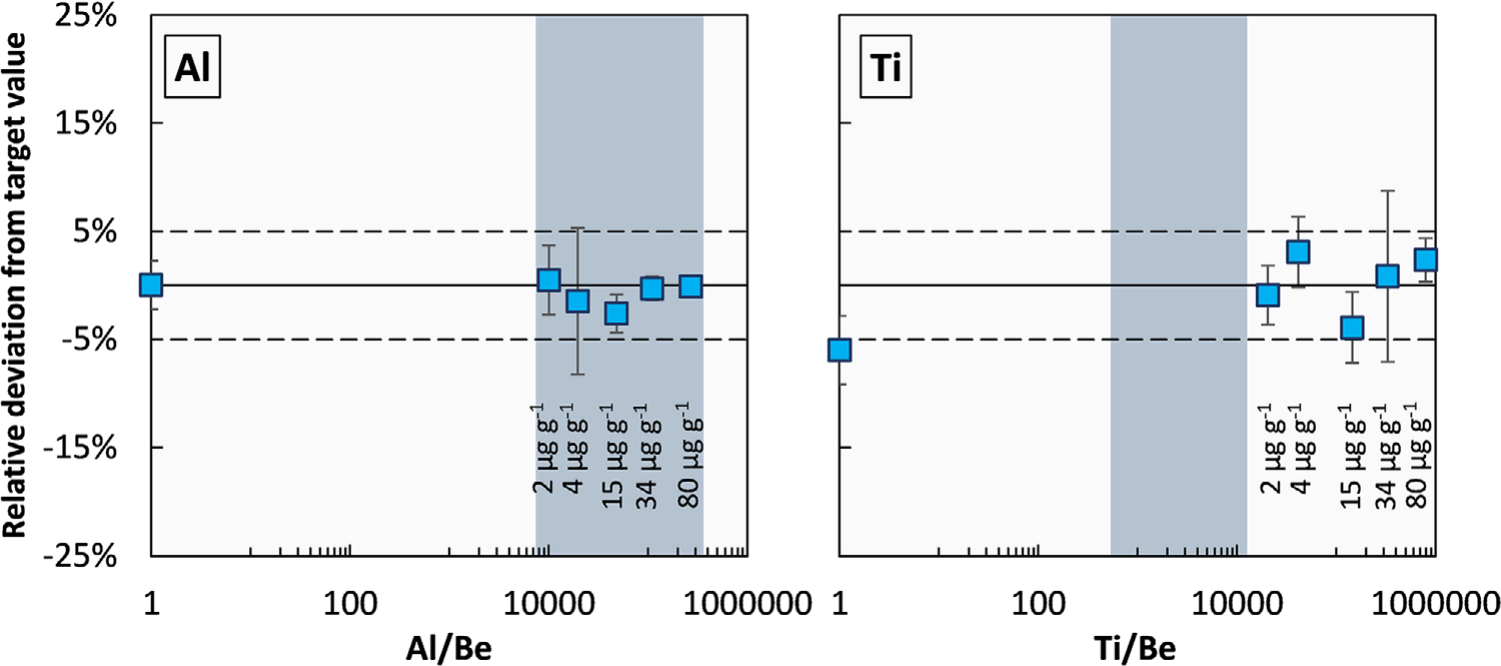
Pure Be solutions doped with Al and Ti to levels typically found in plant material as shown by the grey vertical bars to assess its potential to cause matrix effects during Q‐ICP‐MS analyses. Titanium additions exceeded natural ratios. The solid line indicates no deviation of measured Be concentrations from the target value ±5% calibration uncertainty (dashed lines). Concentrations in μg/g denote the concentration of Al or Ti contained in the doped Be solution. The concentration of Be was constant at 0.25 ng/g for the Al doping test and at 0.10 ng/g for the Ti doping test throughout. Error bars denote the relative standard deviation of three replicates and are lower than the symbol size if not displayed

Triple‐charged elements such as Al and Ti co‐elute with Be (Figure 1). To assess whether either element causes matrix effects during Q‐ICP‐MS analyses, pure Be solutions were doped with Al and Ti in increasing amounts, yielding Al/Be and Ti/Be ratios that are typically found in plants and even exceed natural ratios (Figure 2). Q‐ICP‐MS results show that even for unnatural high ratios of Ti/Be matrix effects are not observed (Figure 2) indicating that further purification with respect to Al and Ti is not required.

### 4.2 Beryllium concentration analyses

The 10‐point calibration curve for Li‐normalized Be ranged typically from 0.02 to 10 ng/g. The limit of detection derived from three times the standard deviation of five to ten consecutive acid blanks (0.3 M HNO_3_ used for dilution) plus the concentration of the blank was 3 pg/g that is typical for Q‐ICP‐MS analyses (eg, see ref.^12^). The limit of quantification derived from ten times the standard deviation of five to ten consecutive acid blanks plus the concentration of the blank was 36 pg/g. The procedural blank, which includes digestion and cation‐exchange chromatography, was below the calibration limit and estimated to amount to a maximum of 9 pg, which corresponds to a typical blank contribution in the order of 0.1% to 1%.

### 4.3 Beryllium concentrations in reference materials

To verify the newly developed method for the analyses of Be concentrations in plant material seven reference materials were selected. Four reference materials are from Wageningen Evaluating Programs for Analytical Laboratories (WEPAL) for which consensus values (N = 16, RSD < 25%), indicative values (N = 8‐16, RSD: 25‐50%), and informative values (N < 8, RSD > 50%) of Be concentrations in ng/g are provided: WEPAL IPE 100 Grass (20 ± 3.0), WEPAL IPE 151 Grass (24 ± 9.0), WEPAL IPE 176 Reed (330 ± 68), and WEPAL IPE 220 Willow wood (4.3 ± 0.14); two reference materials are from the National Institute of Standards & Technology (NIST) for which Be concentrations are not certified: NIST SRM 1515 Apple leaves (no data) and NIST SRM 1573a Tomato leaves (13 ± 2.2 ng/g; see ref.^64^); and one reference material from European Reference Materials (ERM) for which the Be concentration is also not certified: ERM‐CD 281 Rye grass. The provided uncertainties (RSD) on Be concentrations for the reference materials are, apart from IPE 220 Willow wood with ∼3%, relatively high and range from 15% to 38%.

**FIGURE 3:**
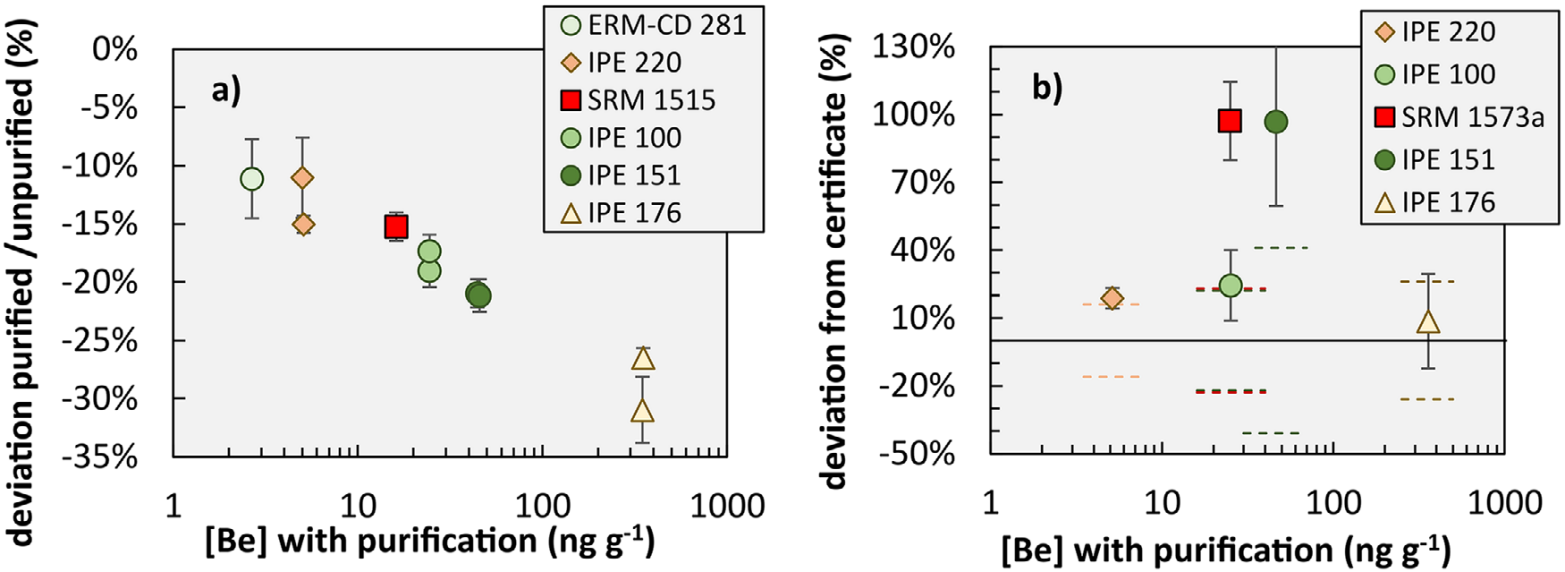
Beryllium concentrations in reference materials. Panel (a): Beryllium concentrations measured with purification (using Li as internal standard) and without purification (using Rh as internal standard) on aliquots of the same plant digest and shown as their relative deviation plotted against Be concentrations with purification. Error bars denote error propagated relative standard deviations from three Be measurement replicates on purified and unpurified samples. Panel (b): Beryllium concentrations after purification plotted against the deviation from certified (IPE samples) or previously published (SRM 1573a) Be concentrations. Error bars denote error propagated relative standard deviations of full analytical replicates and certified RSD´s. Horizontal solid line denote zero deviation. Dashed lines indicate the error propagated long‐term reproducibility of ±16% (determined on 14 full analytical replicates of NIST SRM 1515 with purification) and reported RSD. Dashed lines of IPE 100 and SRM 1573a overlap

Beryllium concentrations in reference materials measured prior and after purification from matrix elements in this study and its respective reported values are summarized in Table S1. The long‐term reproducibility on full analytical replicates (N = 14) in this study is 16% (RSD). Beryllium concentrations of reference materials measured without purification systematically exceed Be concentrations measured after purification (Figure 3a). This result suggests the presence of matrix effects when Be is measured without purification. Mean Be concentrations of reference materials measured after purification match certified and previously reported Be concentrations within the error propagated long‐term reproducibility of 16% (RSD) determined in this study and the reported RSD for most reference materials (Figure 3b). Beryllium concentrations of the reference materials IPE 151 and SRM 1573a measured in this study exceed the indicative value of IPE 151 and the previously reported value of SRM 1573a by about 100%.

### 4.4 Beryllium concentrations in field samples

**FIGURE 4:**
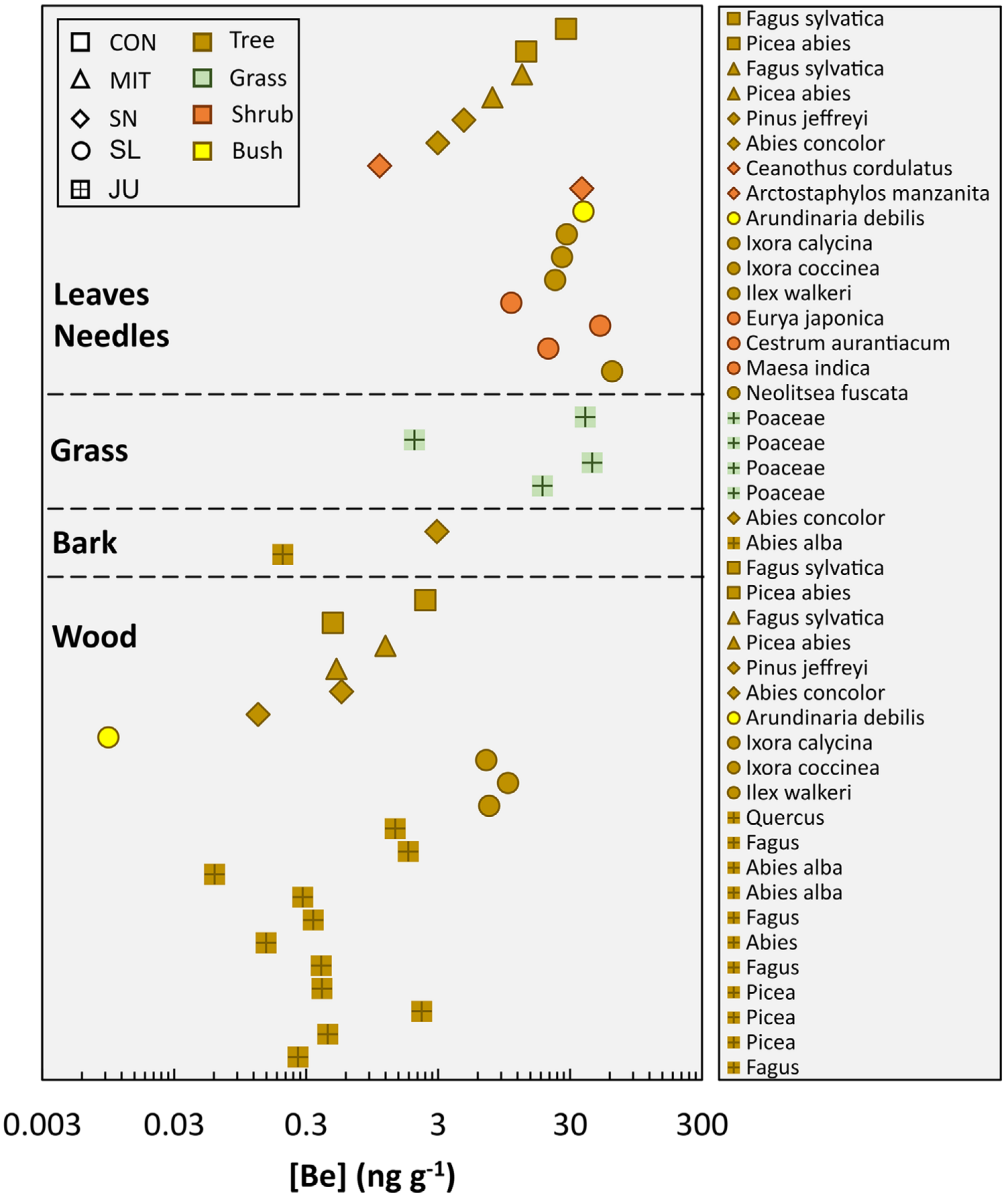
Beryllium concentrations in plants (woody and non‐woody tissue from different tree, shrub and bush species, and grass) from Conventwald (CON), Mitterfels (MIT), Sierra Nevada (SN), Sri Lanka (SL), and Jura (JU). The relative standard deviation of three Be measurement replicates is lower than the symbol size and reported in Table S2

Beryllium concentrations in woody and non‐woody tissue of different tree, shrub, and bush species, and grass at SN, CON, MIT, SL, and JU are shown in Figure 4 and summarized in Table S2. Beryllium concentrations of non‐woody foliage and grass fall into the concentration range of 1.3 to 2000 ng/g previously found in non‐crop plants and surveyed from literature.^1^, ^3^, ^6^, ^7^, ^8^, ^9^, ^10^, ^11^, ^12^, ^13^, ^14^, ^15^ Woody foliage often falls below this concentration range. Whereas Be concentrations in woody tissue range over three orders of magnitude from about 0.01 ng/g to about 10 ng/g, non‐woody foliage varies over two orders of magnitude from about 1.1 ng/g to about 63 ng/g (Figure 4). Grass falls with 2.0 to 44 ng/g within the range of non‐woody foliage and bark falls with 0.2 ng/g to about 3 ng/g within the range found for woody foliage. Beryllium concentrations in non‐woody foliage consistently exceed Be concentrations in woody tissue from the same tree or bush species (Figure 4). Beryllium concentrations are higher in the tropical SL ecosystem than in the temperate areas at SN, CON, MIT, and JU (Figure 4). Moreover, leaves from deciduous trees (eg, *Fagus sylvatica*) and shrubs or bushes have higher Be concentrations than needles from coniferous trees (eg, *Picea abies*, *Abies concolor*, *Pinus jeffreyi*), which is consistent with previously reported data.^65^ Skrivan et al^66^ found a remarkable Be concentration difference in the Czech Republic among wood grown on soil with Be‐rich silicate parent bedrock (13 μg/g^66^) and soil with Be‐poor carbonate parent bedrock (0.38 μg/g^66^). Our results contrast with the result of the Czech Republic study as Be concentrations found in wood grown on soil with Be‐rich silicate parent bedrock (granodiorite at SN: 1.1 ± 0.1 μg/g,^67^ paragneiss at CON: 1.2 ± 0.3 μg/g [unpublished data], paragneiss at MIT: 2.0 ± 0.2 μg/g [unpublished data]) is comparable with wood grown on soil with Be‐poor carbonate parent bedrock (limestone at JU: 0.069 ± 0.044 μg/g (Hella Wittmann, personal communication, 2020)).

## 5 DISCUSSION

### 5.1 Evaluation of the new Be method

Comparison of the newly established Be method with the unpurified sample aliquots on reference materials show that Be concentrations measured without purification consistently exceed Be concentrations measured with purification. This comparison demonstrates either (a) non‐quantitative Be recovery during the purification steps; or (b) the occurrence of matrix effects in the unpurified sample aliquots that are not correctable using Rh as internal standard. Regarding (a), the elution curve using SRM 1515 showed quantitative recovery during the purification (Figure 1). Regarding (b), matrix effects potentially arise because with sample weights of up to 1 g high matrix loads of the sample´s macronutrients K, Ca, and Mg inevitably occur. However, for two reasons we regard the higher matrix load as an unlikely cause of substantial matrix effects. First, macronutrients sum up to a level of about 100 μg/g in the measuring solution, which unlikely saturates the plasma. Second, the presence of K, Ca, and Mg would cause an ionization suppression, which is the opposite effect to the observed intensity increase. Thus, we explore whether residual carbon (C) causes the intensity increase.

**FIGURE 5:**
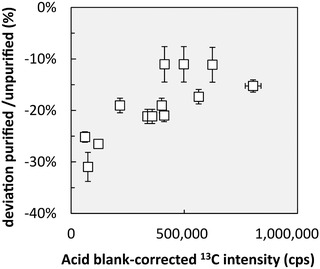
Visualisation of Be intensity increase by residual carbon for reference materials measured in this study prior and after purification of Be from matrix elements. The *Y*‐axis shows the deviation of Be concentrations measured prior and after purification. The *X*‐axis shows the acid blank‐corrected ^13^C intensity measured in reference materials without purification. Error bars of the *X*‐axis denote the RSD of three replicates and error bars of the *Y*‐axis denote error propagated RSD's

Carbon is indeed known to increase the sensitivity of an element, such as Be, that has a slightly lower first ionization energy (Be: 9.3 eV) than carbon (11.26 eV). Here, we argue that this carbon effect is significant in Be concentration analyses without prior purification from matrix elements, because carbon oxidation during microwave‐assisted sample digestion is likely incomplete (see Section 3.4). We used the acid blank‐corrected ^13^C intensity to evaluate the effect of residual carbon on the Be concentration measured without prior purification. Figure 5 shows that C influences Be concentrations measured without purification that is not correctable using Rh as internal standard. Note that the apparent trend in Figure 5 vanishes when removing the three most left‐hand side plotting samples (IPE 176) for which we do not have a reasonable explanation for their positioning on the *Y*‐axis (deviation purified/unpurified). One means to counter the intensity increase effect by C is to dope calibration standards and samples with C at a level at which the sample's remaining C is negligible, for example, by addition of methanol. However, because methanol has a relatively low vapor pressure its fast volatilization adds even more uncertainty. The strength of our new Be method lies thus in its ability to purify Be from residual carbon. An additional benefit is its removal of Li that occurs in trace amounts in plants. This Li, as the optimal internal standard, can be used for Be measurements by Q‐ICP‐MS.

Why the Be concentrations of the reference materials IPE 151 and SRM 1573a measured in this study and their certified and previously published values do not fully agree may be explained by a more complete removal of carbon during microwave digestion in other laboratories than in our study. Alternatively, some of the reference materials may be heterogeneous. Depending on how the laboratories participating on the Wageningen Evaluation Program and Sucharová and Suchara^12^ selected sample weights for plant digestion and dilution factors for Be concentration measurements the matrix elements K, Ca, Mg, and Na may have caused a substantial signal suppression for IPE 151 and SRM 1573a. Noteworthy, the concentrations of the Be signal‐suppressing elements K, Ca, Mg, and Na are more than twofold higher for SRM1573a than for the IPE reference materials. We conclude that the purification of Be from matrix elements with the newly developed method is a real advance in Be concentration analyses in plant materials because it allows the direct comparison of Be concentrations on plant material from different laboratories using different sample digestion methods.

### 5.2 The discrimination of beryllium by plants

**FIGURE 6:**
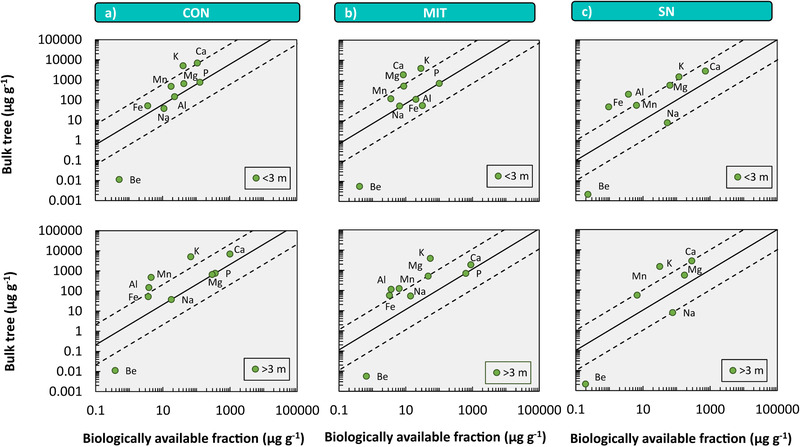
6 Ecological stoichiometry shown by element concentrations of bulk tree *versus* element concentrations of the biologically available fractions above 3 m depth (< 3 m) and below 3 m depth (> 3 m) at Conventwald (CON, panel A), Mitterfels (MIT, panel B) and Sierra Nevada (SN, panel C). Bulk tree element concentrations were estimated by averaging the concentrations from woody and non‐woody tissue of the same tree species. At CON and MIT, the displayed bulk tree concentration represents an average of bulk *Picea abies* and bulk *Fagus sylvatica*. At SN the bulk *Pinus jeffreyi* is shown. Al, Fe, and P that are shown in panel (A) and (B) but only in part or not in panel (C) were below limit of detection. All data but Be concentrations for CON and MIT were taken from Uhlig and von Blanckenburg ^57^ and for SN from Uhlig et al^68^; Be concentrations of the biologically available fraction for SN is taken from Dannhaus ^67^. Solid line illustrates the 1:1 line, dashed lines illustrate 1:10 and 10:1 lines. 1:1 lines are forced through P at CON and MIT where biologically available P concentrations are known because P is assumed to follow ideal ecological stoichiometry

To evaluate whether Be is beneficial or essential to plants, we tested whether Be concentrations in plants and other plant essential and beneficial elements correlate with those found in the biologically available fraction of elements in soil and the weathered rock beneath it (see Section 3). In doing so, Be concentrations were added to such correlations for CON and MIT (Figure 6), and for SN (Figure 6) but not for SL and JU due to the lack of data on the biologically available fraction. The bulk tree composition was estimated following Uhlig and von Blanckenburg^42^ by averaging the concentrations from woody and non‐woody tissue of the same tree species. Whereas most elements plot along the 1:1 line (forced through the often plant growth limiting mineral nutrient P) plus or minus one order of magnitude Be concentrations in plants are about two orders of magnitude lower than in the biologically available fraction (Figure 6). This discrepancy may be attributed to three reasons: (a) the Be concentration in bulk tree was underestimated, (b) the Be concentration in the biologically available fraction is overestimated; and (c) forest trees actively discriminate against Be uptake to prevent toxicity. Regarding (a), an underestimate is possible if the bulk tree´s Be is mainly located in roots that were not sampled in this study. Indeed, Romney and Childress^33^ showed that Be concentrations are highest in roots and Kaplan et al^24^ showed that up to 97% of Be utilized by food crops remains in roots. However, as explained in Section 1, we regard this inference to result from conducting experiments above the solubility limit of Be. Regarding (b), an overestimate of the Be concentration in the biologically available fraction is feasible because the Be extraction method differs from those of the other elements (see Section 3.7), namely from the amorphous Fe‐oxyhydroxide fraction extracted with 0.5 M HCl rather than with 1 M HCl for P and 1 M NH_4_OAc used for the other elements. We regard this as unlikely because this fraction is known to exchange Be readily with aqueous solutions^4^, ^5^ and also because unrealistically low Be concentrations of <0.01 μg/g would need to prevail in that fraction (Figure 6, by extrapolation). For that reason, Be is most likely actively discriminated from uptake by trees into shoots, but also shrubs and bushes to prevent toxicity that ultimately may result in mortality.

Beryllium concentrations in plants have the potential to serve as a bioindicator of environmental Be pollution.^36^ Given that Be is highly carcinogenic, this application may be of importance to agriculture. Even though trees, shrubs, and bushes sampled in this study represent only a small part of the floral diversity, these samples originate from Be non‐polluted environments. Thus, Be concentrations of bulk plants or individual plant organs sampled in this study (we exclude literature data here because it is rarely reported whether field sites are Be polluted) are useful to suggest a natural baseline of Be concentrations in plants of about 52 ng/g estimated from the 95 percentile of non‐woody tissue measured in this study in Be non‐polluted environments. However, Be concentrations in field crops on Be non‐polluted sites are needed to further develop Be concentrations in plants to a promising bioindicator for Be polluted sites.

## 6 CONCLUSIONS

The advantages of the new method for Be concentration analysis in plant material are (a) minimizing potential signal suppression of Be by matrix elements, (b) eliminating sensitivity increases caused by remaining carbon after microwave assisted plant digestion, and (c) allowing the usage of Li as optimal internal standard for Be to monitor and correct for potential sensitivity fluctuations during Q‐ICP‐MS measurements. Verification of the new method by processing seven reference materials of different plant matrices shows that Be concentrations measured prior purification from matrix elements exceed systematically those measured after purification due to sensitivity increases caused by remaining carbon. Given the advantages of the new method and different plant digestion routines in different laboratories, we suggest that the pre‐purification method should routinely be used in future plant studies. From applying the new method to field samples from temperate forests and a tropical rainforest, we suggest a natural baseline of Be concentrations in plants of about 52 ng/g. This baseline may be applicable to use Be concentrations in plants as a bioindicator for Be pollution in the environment. Finally, comparison of Be concentrations in plants with the concentration in the biologically available fraction of the soil reveals that Be is most likely actively discriminated against uptake to prevent toxicity.

## CONFLICTS OF INTEREST

The authors declare that they have no conflict of interest.

## Supporting information

Supporting Information
