# Supplementary material for: Quantifying beryllium concentrations in plant shoots from forest ecosystems using cation‐exchange chromatography and quadrupole ICP‐MS
Source: Anal Sci Adv. 2020 Jun 8;1(1):8–21. doi: 10.1002/ansa.202000036 (PMC10989106; doi:10.1002/ansa.202000036)
Supplement: Supplementary file 1 — Supporting Information [file ANSA-1-8-s001.pdf]

ANALYTICAL SCIENCE ADVANCES

Analytical Science Advances

Supporting Information for
doi.org/10.1002/ansa.202000036

Quantifying beryllium concentrations in plant shoots from forest ecosystems using cation-exchange chromatography and quadrupole ICP-MS

David Uhlig[1*], Tatiana Goldberg[1], Daniel A. Frick[1], Friedhelm von Blanckenburg[1,2]

[1] GFZ German Research Centre for Geosciences, Section 3.3 Earth Surface Geochemistry, Telegrafenberg, 14473 Potsdam, Germany
* present address: Institute of Bio- and Geosciences (IBG-3) Agrosphere, Forschungszentrum Jülich, Wilhelm-Johnen-Str, 52425 Jülich, Germany, d.uhlig@fz-juelich.de
[2] Freie Universität Berlin, Institute of Geological Sciences, Malteserstr. 74-100, 12249 Berlin, Germany

Content of this file

- Table S1 Beryllium concentrations in plant standard reference materials
- Table S2 Beryllium concentrations in plant field samples

Table S1 Beryllium concentrations in plant standard reference materials.

**sample ID**	**Be (ng g^{-1})**	**RSD (%)**	**Be (ng g^{-1})**	**RSD (%)**
	with purification		no purification	
ERM-CD 281 Rye grass (1)	2.9	2.4	n.d.	n.d.
ERM-CD 281 Rye grass (2)	3.0	2.5	n.d.	n.d.
ERM-CD 281 Rye grass (3)	3.5	1.9	n.d.	n.d.
ERM-CD 281 Rye grass (4)	2.7	0.76	3.0	3.3
**ERM-CD 281 Rye grass mean**	**3.0**		**3.0**	
ERM-CD 281 Rye grass SD	*0.34*		-	
ERM-CD 281 Rye grass N	*4*		*1*	
ERM-CD 281 Rye grass RSD (%)	*11%*		-	
NIST SRM 1515 Apple Leaves (1)	24	0.27	n.d.	n.d.
NIST SRM 1515 Apple Leaves (2)	24	1.0	n.d.	n.d.
NIST SRM 1515 Apple Leaves (3)	24	0.41	n.d.	n.d.
NIST SRM 1515 Apple Leaves (4)	18	0.20	n.d.	n.d.
NIST SRM 1515 Apple Leaves (5)	18	2.7	n.d.	n.d.
NIST SRM 1515 Apple Leaves (6)	18	3.0	n.d.	n.d.
NIST SRM 1515 Apple Leaves (7)	19	1.0	n.d.	n.d.
NIST SRM 1515 Apple Leaves (8)	19	0.45	n.d.	n.d.
NIST SRM 1515 Apple Leaves (9)	25	3.0	n.d.	n.d.
NIST SRM 1515 Apple Leaves (10)	18	3.1	n.d.	n.d.
NIST SRM 1515 Apple Leaves (11)	15	1.7	n.d.	n.d.
NIST SRM 1515 Apple Leaves (12)	17	0.46	n.d.	n.d.
NIST SRM 1515 Apple Leaves (13)	16	0.27	19	1.2
NIST SRM 1515 Apple Leaves (14)	17	n.a.	n.d.	n.d.
**SRM 1515 Apple leaves mean**	**19**		**19**	
SRM 1515 Apple leaves SD	*3.2*		-	
SRM 1515 Apple leaves N	*14*		*1.0*	
SRM 1515 Apple leaves RSD (%)	*16%*		-	
NIST SRM 1573a Tomato Leaves (1)	25	n.a.	n.d.	n.d.
NIST SRM 1573a Tomato Leaves GeoRem value	13		-	
NIST SRM 1573a Tomato Leaves absolute uncertainty	*2.2*		-	
NIST SRM 1573a Tomato Leaves N	*1*		-	
NIST SRM 1573a Tomato Leaves relative uncertainty (%)	*17%*		-	
**relative difference (%)**	97%		-	
WEPAL IPE 151 Grass / Poaceae (1)	44	0.65	56	1.0
WEPAL IPE 151 Grass / Poaceae (2)	46	0.20	58	1.4
WEPAL IPE 151 Grass / Poaceae (3)	49	n.a.	n.d.	n.d.
**WEPAL IPE 151 Grass / Poaceae mean**	**46**		**57**	
WEPAL IPE 151 Grass / Poaceae SD	*2.5*		*1.2*	
WEPAL IPE 151 Grass / Poaceae N	*3*		*2*	
WEPAL IPE 151 Grass / Poaceae RSD (%)	*5%*		*2%*	
WEPAL IPE 151 Grass / Poaceaes indicative value	24		-	
WEPAL IPE 151 Grass / Poaceae absolute uncertainty	*9.0*		-	
WEPAL IPE 151 Grass / Poaceae N	*8*		-	
WEPAL IPE 151 Grass / Poaceae relative uncertainty (%)	*38%*		-	
**relative difference (%)**	97%		142%	
WEPAL IPE 220 Willowwood / Salicaceae (1)	5.0	0.3	5.6	3.4
WEPAL IPE 220 Willowwood / Salicaceae (2)	5.1	0.7	6.0	0.24
WEPAL IPE 220 Willowwood / Salicaceae (3)	5.3	n.a.	n.d.	n.d.
**WEPAL IPE 220 Willowwood / Salicaceae mean**	**5.1**		**5.8**	
WEPAL IPE 220 Willowwood / Salicaceae SD	*0.16*		*0.25*	
WEPAL IPE 220 Willowwood / Salicaceae N	*3*		*2*	
WEPAL IPE 220 Willowwood / Salicaceae RSD (%)	*3%*		*4%*	
WEPAL IPE 220 Willowwood / Salicaceae informative value	4.3		-	
WEPAL IPE 220 Willowwood / Salicaceae absolute uncertainty	*0.14*		-	
WEPAL IPE 220 Willowwood / Salicaceae N	3		-	
WEPAL IPE 220 Willowwood / Salicaceae relative uncertainty (%)	3%		-	
**relative difference (%)**	19%		34%	
WEPAL IPE 100 Grass (gr94) / Poaceae (1)	24	0.45	30	1.4
WEPAL IPE 100 Grass (gr94) / Poaceae (2)	24	0.10	30	1.4
WEPAL IPE 100 Grass (gr94) / Poaceae (3)	27	n.a.	n.d.	n.d.
**WEPAL IPE 100 Grass (gr94) / Poaceae mean**	**25**		**30**	
WEPAL IPE 100 Grass (gr94) / Poaceae SD	*1.4*		*0.46*	
WEPAL IPE 100 Grass (gr94) / Poaceae N	*3*		*2*	
WEPAL IPE 100 Grass (gr94) / Poaceae RSD (%)	*5%*		*2%*	
WEPAL IPE 100 Grass (gr94) / Poaceae consensus value	20		-	
WEPAL IPE 100 Grass (gr94) / Poaceae absolute uncertainty	3.0		-	
WEPAL IPE 100 Grass (gr94) / Poaceae N	24		-	
WEPAL IPE 100 Grass (gr94) / Poaceae relative uncertainty (%)	15%		-	
**relative difference (%)**	24%		47%	

continued next page …

Table S1 continued Beryllium concentrations in plant standard reference materials.

WEPAL IPE 176 Reed / Phragmites communis (1)	348	2.6	504	1.0
WEPAL IPE 176 Reed / Phragmites communis (2)	352	0.38	479	0.78
WEPAL IPE 176 Reed / Phragmites communis (3)	375	0.16	501	1.0
**WEPAL IPE 176 Reed / Phragmites communis mean**	**358**		**495**	
WEPAL IPE 176 Reed / Phragmites communis SD	*14*		*14*	
WEPAL IPE 176 Reed / Phragmites communis N	*3*		*3*	
WEPAL IPE 176 Reed / Phragmites communis RSD (%)	*4%*		*3%*	
WEPAL IPE 176 Reed / Phragmites communis indicative value	330		-	
WEPAL IPE 176 Reed / Phragmites communis absolute uncertainty	68		-	
WEPAL IPE 176 Reed / Phragmites communis N	9		-	
WEPAL IPE 176 Reed / Phragmites communis relative uncertainty (%)	20%		-	
**relative difference (%)**	9%		50%	

RSD = relative standard deviation
n.a. = not available because only one main run was measured; n.d. = not determined

Table S2 Beryllium concentrations in plant field samples.

sample ID	IGSN †	species name	plant family	compartment	Be (ng g^{-1})	RSD (%)
Sierra Nevada (SN)						
SN WHL	GFFB10032	*Ceanothus cordulatus*	*Rhamnaceae*	Shrub leaves	37	0.03
SN ML	GFFB10031	*Arctostaphylos manzanita*	*Ericaceae*	Shrub leaves	1.1	0.87
SN JPN	GFFB10030	*Pinus jeffreyi*	*Pinaceae*	Tree needles	4.7	1.5
SN JPS	GFFB10034	*Pinus jeffreyi*	*Pinaceae*	Tree branch	0.56	0.80
SN WFB	GFJUB0012	*Abies concolor*	*Pinaceae*	Tree bark	2.9	0.64
SN WFL	GFJUB0011	*Abies concolor*	*Pinaceae*	Tree needles	3.0	0.61
SN WFS	GFJUB0013	*Abies concolor*	*Pinaceae*	Tree branch	0.13	n.a.
Conventwald (CON)						
CON V 3	GFDUH00Q7	*Fagus sylvatica*	*Fagaceae*	Tree leaves	28	0.42
CON V 6,7	GFDUH00TG	*Fagus sylvatica*	*Fagaceae*	Tree stemwood	2.4	0.69
CON V 4	GFDUH00Q8	*Picea abies*	*Pinaceae*	Tree needles	14	0.43
CON V 14.15	GFDUH00TH	*Picea abies*	*Pinaceae*	Tree stemwood	0.48	0.93
Mitterfels (MIT)						
MIT V 9	GFDUH006W	*Fagus sylvatica*	*Fagaceae*	Tree leaves	13	0.53
MIT V 14,15,16	GFDUH00TL	*Fagus sylvatica*	*Fagaceae*	Tree stemwood	1.2	0.59
MIT V 11	GFDUH006S	*Picea abies*	*Pinaceae*	Tree needles	7.8	0.51
MIT V 23.25	GFDUH00TK	*Picea abies*	*Pinaceae*	Tree stemwood	0.51	0.84
Sri Lanka (SL)						
SL BBB	GFFB10069	*Arundinaria debilis*	*Gramineae*	Bush leaves	38	n.a.
SL V 6	GFFB10065	*Arundinaria debilis*	*Gramineae*	Bush twigs	0.01	2.8
SL V 11	GFFB10068	*Ixora calycina*	*Rubiaceae*	Small tree leaves	28	2.8
SL V 7	GFFB10066	*Ixora calycina*	*Rubiaceae*	Small tree twigs	7.0	2.0
SL V 4a	GFFB1005Z	*Ilex walkeri*	*Myrtaceae*	Small tree leaves	23	2.2
SL SOB	GFFB10060	*Ilex walkeri*	*Myrtaceae*	Small tree twigs	7.3	n.a.
SL V 2	GFJUB0062	*Ixora coccinea*	*Rubiaceae*	Small tree leaves	26	0.80
SL V 3	GFFB10064	*Ixora coccinea*	*Rubiaceae*	Small tree branch	10	0.98
SL V 1	GFFB10067	*Cestrum aurantiacum*	*Solanaceae*	Shrub leaves	51	0.94
SL GL	GFFB10061	*Eurya japonica*	*Theaceae*	Shrub/ small tree leaves	11	2.7
SL HL	GFFB10062	*Maesa indica*	*Myrsinaceae*	Shrub leaves	21	0.15
SL V 5B	GFFB10063	*Neolitsea fuscata*	*Lauraceae*	Tree leaves	63	0.32
Jura (JU)						
Dard						
JU17 05 V01	GFHWO000K	*Quercus*	*Fagaceae*	Tree stemwood	1.4	0.87
JU17 05 V03	GFHWO000L	*Fagus*	*Fagaceae*	Tree stemwood	1.8	0.81
Doubs						
JU17 10a V04	GFHWO000M	*Abies alba*	*Pinaceae*	Tree stemwood	0.06	2.8
JU17 10a V05	GFHWO000N	*Abies alba*	*Pinaceae*	Tree bark	0.20	4.4
JU17 10a V06	GFHWO000P	*Abies alba*	*Pinaceae*	Tree stemwood	0.28	2.5
JU1707bV01	GFHWO000T	*Fagus*	*Fagaceae*	Tree stemwood	0.34	1.7
JU1707bV02	GFHWO000U	*Abies*	*Pinaceae*	Tree stemwood	0.15	6.1
Saine						
JU1710aV12	GFHWO000Q	*Fagus*	*Fagaceae*	Tree stemwood	0.39	2.0
JU1706cV01	GFHWO000S	*Picea*	*Piceoideae*	Tree stemwood	0.40	1.9
Dessoubre						
JU1708V06	GFHWO000V		*Poaceae*	Grass	39	1.5
Dahon						
JU1709aV02	GFHWO000W		*Poaceae*	Grass	2.0	1.0
JU17 09b V03	GFHWO000H		*Poaceae*	Grass	44	1.3
JU1709V02	GFHWO000X	*Picea*	*Piceoideae*	Tree stemwood	2.3	0.58
JU1709bV07	GFHWO000G	*Picea*	*Piceoideae*	Tree stemwood	0.44	1.2
Lison						
JU1711bV01	GFHWO000J		*Poaceae*	Grass	19	2.1
JU1711cV01	GFHWO000R	*Fagus*	*Fagaceae*	Tree stemwood	0.26	1.2

RSD = relative standard deviation
n.a. = not available because only one main run was measured
† IGSN (International Geo Sample Number). Metadata of samples are available under: www.igsn.org by adding the IGSN after igsn.org, e.g. igsn.org/GFFB10031
